# Linking human male vocal parameters to perceptions, body morphology, strength and hormonal profiles in contexts of sexual selection

**DOI:** 10.1038/s41598-020-77940-z

**Published:** 2020-12-04

**Authors:** Christoph Schild, Toe Aung, Tobias L. Kordsmeyer, Rodrigo A. Cardenas, David A. Puts, Lars Penke

**Affiliations:** 1 Department of Psychology, University of Copenhagen, Øster Farimagsgade 2A, 1353 Copenhagen, Denmark; 2 Department of Anthropology and Center for Brain, Behavior and Cognition, Pennsylvania State University, University Park, PA 16802 USA; 3 Department of Psychology and Leibniz ScienceCampus Primate Cognition, University of Goettingen, Gosslerstrasse 14, 37073 Göttingen, Germany; 4 Department of Psychology, Pennsylvania State University, University Park, PA 16802 USA

**Keywords:** Evolution, Evolutionary theory, Sexual selection, Human behaviour

## Abstract

Sexual selection appears to have shaped the acoustic signals of diverse species, including humans. Deep, resonant vocalizations in particular may function in attracting mates and/or intimidating same-sex competitors. Evidence for these adaptive functions in human males derives predominantly from perception studies in which vocal acoustic parameters were manipulated using specialist software. This approach affords tight experimental control but provides little ecological validity, especially when the target acoustic parameters vary naturally with other parameters. Furthermore, such experimental studies provide no information about what acoustic variables indicate about the speaker—that is, why attention to vocal cues may be favored in intrasexual and intersexual contexts. Using voice recordings with high ecological validity from 160 male speakers and biomarkers of condition, including baseline cortisol and testosterone levels, body morphology and strength, we tested a series of pre-registered hypotheses relating to both perceptions and underlying condition of the speaker. We found negative curvilinear and negative linear relationships between male fundamental frequency (*f*_o_) and female perceptions of attractiveness and male perceptions of dominance. In addition, cortisol and testosterone negatively interacted in predicting *f*_o_, and strength and measures of body size negatively predicted formant frequencies (*P*_*f*_). Meta-analyses of the present results and those from two previous samples confirmed that *f*_o_negatively predicted testosterone only among men with lower cortisol levels. This research offers empirical evidence of possible evolutionary functions for attention to men’s vocal characteristics in contexts of sexual selection.

## Introduction

Acoustic signals comprise a fundamental component of mating competition^1–4^ and are highly sexually dimorphic in many species, including many anthropoid primates. Humans in particular exhibit strong sexual dimorphism in acoustic signals^5^, such that the distributions of male and female vocal parameters related to pitch and timbre barely overlap^6^.

From hearing the voice alone, humans can assess diverse salient social characteristics of a speaker, such biological sex, age and physical strength^7–9^. Many of these evaluations rely on inter-individual variation in specific sets of vocal parameters, including fundamental frequency and formant frequencies^5,10^. Fundamental frequency (*f*_o_) is the rate of vocal fold vibration during phonation and influences perceptions of pitch. Formant frequencies are resonant frequencies determined by the length and shape of the vocal tract and influence perceptions of vocal timbre.

Fundamental and formant frequencies are some of the most sexually dimorphic characteristics in humans, suggesting a past influence of sexual selection^11^. Indeed, lower male *f*_o_ predicts greater perceptions of attractiveness, dominance and masculinity^12–14^, as well as greater mating success^14,15^ (but see^16^ for a null finding) and reproductive success^15,17^ (see also^18,19^). Likewise, male formant frequencies influence perceptions of attractiveness, dominance and masculinity^12,13,20,21^.

Despite the abundance of evidence linking acoustic parameters to perceptions relevant in mating competition, a fundamental question remains: Why have humans evolved to attend to these parameters? Costly signaling theory (originally proposed by^22,23^, but see^24^) which concerns the transmission of reliable information between signalers and receivers, is a useful theoretical tool to answer this question and helps us understand the maintenance of signal honesty via receiver-independent (production costs, developmental costs, maintenance costs) and receiver-dependent costs (e.g., retaliation costs, vulnerability costs; see^25,26^ for reviews). Recently, some authors^27,28^ have pointed out weak receiver-independent costs associated with men’s *f*_o_ and concluded that men’s *f*_o_ does not signal formidability. Others^29–31^ suggest that men’s *f*_o_ is likely to be partly honest.

Although *f*_o_ influences perceptions of physical dominance, it correlates only weakly with physical strength^6,9,32^ (see^29^ for a meta-analysis) and body height^33^. Past research also points to associations with hormonal profiles in males: *f*_o_ decreases strongly during, and higher circulating testosterone levels predict lower *f*_o_ in men^11,34,35^ (see^29^ for a meta-analysis). Further, the relationship between *f*_o_ and testosterone was found to be stronger in men with lower cortisol levels^5^, a pattern that has been associated with immunocompetence^36^. Another study^37^ that utilized salivary immunoglobulin-A (sIgA; a marker of mucosal immunity) as a measure of immunocompetence reported that sIgA was negatively correlated with *f*_o_. In a similar vein, listeners assigned higher dominance ratings, but not higher health ratings, to speakers with higher self-reported health^38^. Overall, these studies suggest, that *f*_o_ may be a partly honest signal of condition^29–31^. Formants are closely tied to vocal tract length and are therefore indirect, albeit weak, correlates of body size in humans^33,39,40^. Additionally, a recent study showed significant correlations with other somatometric measures, such as body mass index and hip circumference^41^. However, links between formants and physical strength are equivocal^6,32^.

In addition to the paucity of evidence concerning the information content of male voices, there are also significant gaps in knowledge concerning how men’s voices may influence social perceptions. For example, because most prior studies manipulated only one acoustic parameter at a time in experimental settings, the relative importance of different parameters in forming social judgments have not been well characterized. Prior research also has primarily investigated linear relationships (Table 1), and thus it remains largely unknown whether acoustic parameters have curvilinear effects on perceptions, which have been predicted in some cases^11^. Vocal stimuli in most prior work are also unnaturally invariant in content and motivation, with all speakers uttering a series of vowels, counting, or speaking precisely the same, often socially irrelevant, phrase; hence, the generalizability and external validity of such results depend on whether the effects they reveal persist in natural speech^13^. Finally, only a few, mostly low-powered studies (Table 1) have simultaneously shown that these acoustic parameters are related to both perceptions of attractiveness and/or dominance on the one hand and indirect measures of mate quality and formidability on the other.

**Table 1:** A non-exhaustive list of studies (n = 50) on human voice perception.

No	Studies	Rater (n)	Vocalizers (n)	Perceptions evaluated	Vocalizer's condition	Natural voices	Cuvilinear tested
1	Schild et al.^42^	95	181	Trus	Trus	+	+
2	Collins and Missing^43^	30	30	Att; Age	Size	+	
3	Puts et al.^5^	1126	548	Att; Dom	T; C	+	
4	Raine et al.^44^	150	61	Size	Size	+	
5	Raine et al.^45^	135	61	Size	Size	+	
6	Rendall et al.^46^	163	68	Size	Size	+	
7	Rosenfield et al.^15^	84	4	Att; Pres; Dom	MS	+	
8	Šebesta et al.^47^	62	93	Att	Size	+	
9	Šebesta et al.^48^	63	40	Dom	Size	+	
10	Simmons et al.^49^	30	44	Att; Mas	Semen	+	
11	Valentova et al.^50^	203	152	Att	Size	+	
12	Armstrong et al.^27^	224	183	Dom; Size	Size	+	
13	Feinberg et al.^51^	991	123	Age; Att; Fem		+	+
14	Babel et al.^52^	30	60	Att		+	
15	Gregory et al.^53^	118	60	Com Qual		+	
16	Hodges-Simeon et al.^13^	330	111	Att; Dom		+	
17	Knowles et al.^54^	180	32	Cop		+	
18	Michalsky and Schoormann^55^	20	20	Att; Like		+	
19	Pisanski and Rendall^56^	129	89	Size; Att; Mas; Fem		+	
20	Pisanski et al.^57^	68	20	Size; Att; Mas; Fem		+	
21	Sorokowski et al.^58^	39	51	Comp; Auth		+	
22	Valentova et al.^59^	84	30	Att; Mas		+	
23	Hill et al.^60^	1349	471	Att	Fac Sym		+
24	Wolff and Puts^61^	376	117	Dom	Size; T; Agg		+
25	Shirazi et al.^62^	128	6	Att	E; P		
26	Re et al.^63^	19	64	Att; Mas; Fem			+
27	Saxton et al.^64^	40	6	Att; Dom			+
28	Apicella and Feinberg^65^	88	10	Att			
29	Borkowska and Pawlowski^66^	473	58	Att; Dom			
30	Bruckert et al.^67^	64	55	Att			
31	Feinberg et al.^68^	68	5	Att; Dom			
32	Feinberg et al.^69^	26	8	Att; Dom			
33	Feinberg et al.^70^	1759	6	Pref			
34	Feinberg et al.^71^	83	6	Att			
35	Fraccaro et al.^72^	179	8	Att; Dom			
36	Hughes et al.^73^	40	40	Att			
37	Jones et al.^74^	800	12	Att; Dom			
38	Klofstad et al.^75^	382	27	Com; Size; Trus			
39	Leaderbrand et al.^76^	48	4	Att			
40	O'Connor et al.^77^	138	6	Att; Inv			
41	Puts et al.^78^	86	111	Dom			
42	Puts et al.^20^	42	30	Dom			
43	Puts et al.^79^	109	4	Att; Flir			
44	Puts^14^	142	111	Att			
45	Riding et al.^80^	54	9	Att			
46	Suire et al.^81^	225	58	Att			
47	Tigue et al.^82^	165	15	Int; Prow; Vote			
48	Vukovic et al.^83^	70	6	Att; Dom; Trus			
49	Watkins et al.^84^	50	10	Dom			
50	Xu et al.^85^	42	2	Att; Emo			

A list of 50 studies that relate to mating-relevant perceptions of human voice was obtained via Google Scholar search. Most studies that investigate human voice perceptions tested only on perceptions (n = 35), used manipulated voice stimuli (n = 28), and tested linear relationships (n = 44). Agg = Aggressiveness; Att = Attractiveness; C = Cortisol; Com = Competent; Com Qual = Communication Quality; Cop = Cooperativeness; Dom = Dominance; Emo = Emotions; E = Estradiol; Fac Sym = Facial Symmetry; Flir = Flirtatiousness; Fem = Femininity; Int = Integrity; Inv = Investing; Mas = Masculinity; MS = Mating Success; P = Progesterone; Pref = Preference; Pres = Prestige; Prow = Prowess; T = Testosterone; Trus = Trustworthiness; +  = Presence.

Given the fundamental gaps in knowledge outlined above, we conducted a preregistered study (preregistration: https://osf.io/nrmpf/) to examine (1) how vocal parameters are utilized in assessing dominance and attractiveness, and (2) why using those parameters for judgments could be adaptive insofar as they are associated with indirect measures of mate quality and/or formidability. In contrast to most studies on perceived vocal attractiveness and dominance, which have used standardized voice samples (i.e. counting, vowels or standardized passages), more natural stimuli were used to augment external validity. Importantly, we use a relatively large (N = 160) and rich dataset, which allows relationships between vocal parameters, baseline cortisol and testosterone levels, body morphology and strength to be tested in a single sample.

### Perceptions of attractiveness and dominance

Because deep male voices may display social power^29^, threat potential^11^, and predict greater anticipated^42,86,87^ and actual^42,88^ sexual infidelity, there may be costs as well as benefits to mating with males with masculine voices^11^. Further, some studies suggest that the link between mean *f*_o_ and attractiveness is weaker and rather curvilinear: Very low-pitched voices are not seen as more attractive and sometimes even less attractive as low-pitched voices^11,64^. In line with the context-dependent nature of costs and benefits and reports from previous literature, we therefore predicted negative linear^5^ and negative quadratic^11^ relationships between attractiveness ratings and both mean *f*_o_ (**H1**) and formant position (*P*_*f*_) (**H2**). *P*_*f*_ is a measure of formant structure, calculated as the average standardized formant value for the first *n* (usually four) formants^6^.

Masculine voices (i.e. low *f*_o_ and *P*_*f*_) have been found to be preferred by females to a greater extent in short-term compared to long-term relationship contexts^14,89^. This might reflect an adaptive trade-off strategy in which a mate’s genetic fitness, putatively indicated by masculine traits, is granted greater value in short-term contexts, whereas his expected investment and fidelity are valued more in long-term contexts^89,90^. Consequently, we predicted stronger relationships between short-term, compared to long-term, attractiveness ratings and both mean *f*_o_ (**H3**) and *P*_*f*_ (**H4**).

It has been hypothesized that deep voices display threat potential^6^; hence, we predicted negative relationships between dominance ratings and both mean *f*_o_ (**H5**) and *P*_*f*_ (**H6**). According to the source-filter theory, *f*_o_ and *P*_*f*_ are theoretically distinct^91^. They are also only weakly correlated^10^ and seem to convey different information about a male speaker^6^. Accordingly, we predicted *f*_o_ and *P*_*f*_ to be independent predictors of both attractiveness (**H7**) and dominance (**H8**) ratings.

### Indirect measures of mate quality and formidability

Previous studies^34,35^ linked lower *f*_o_ to higher circulating testosterone levels, and more recently this relationship was found to be stronger in men with lower cortisol levels^5^, a result seemingly consistent with the stress-linked immunocompetence handicap hypothesis that *f*_o_ honestly signals a speaker’s physical condition^36^. We therefore predicted a negative relationship between mean *f*_o_ and testosterone (**H9**) and predicted that this relationship would be attenuated by high baseline cortisol (**H10**).

Formants have been shown to relate moderately to body height, a phenotype that is relevant in both intra- and intersexual selective contexts^92^. We therefore predicted a negative relationship between *P*_*f*_ and body height (**H11**).

### Exploratory analyses

In addition to these preregistered predictions, we conducted the following exploratory analyses. First, we examined how vocal parameters related to physical strength and body morphology. Second, we compared whether distinct parameters are used as cues for ratings on social dominance (i.e. being respected) and physical dominance (i.e. fighting ability), as they describe separate aspects of social evaluation^93^. Third, we explored whether jitter and shimmer influence attractiveness and dominance perceptions, as these acoustic parameters seem to provide information on male body shape. Jitter and shimmer quantify cycle-to-cycle variation in *f*_o_ and amplitude, respectively, and influence perceptions of voice roughness. Fourth, we conducted three mediation analyses: (1) a moderated mediation model to test whether *f*_o_ mediates the relationship between vocalizers’ testosterone levels (condition) and dominance ratings (perception), and whether this mediation is further moderated by cortisol, (2) a mediation model to test whether *f*_o_ and *P*_*f*_, mediate the relationship between vocalizers’ height and dominance ratings, and 3) a mediation model to test whether *f*_o_ and *P*_*f*_, mediate the relationship between vocalizers’ composite measure of size (extracted via factor analysis with varimax rotation) and dominance ratings. We conducted a separate mediation model for height, in addition to its inclusion in the factor analysis, as height has been shown to reflect good nutrition and low stress during development, as well as genetic predictors of immune function^94^. Additionally, a recent study^31^ reported that *f*_o_ mediated the relationship between height and physical dominance ratings in two separate samples. Finally, we conducted three meta-analyses to test: (1) the mediating effect of *f*_o_ between height and dominance ratings, (2) whether cortisol and testosterone negatively interact to predict male *f*_o_, and 3) whether *f*_o_ negatively predicts testosterone levels, especially among men with lower cortisol levels.

## Design and methods

### Participants

One hundred sixty-five heterosexual males participated in a study on testosterone reactivity and personality state changes, which was conducted at the University of Goettingen, Germany (for details, see^95^). Each participant provided a standardized video recording, saliva samples, body morphology measurements, and handgrip as well as upper-body strength. Data from five individuals could not be used due to technical issues during video recording or because consent for further use of the video material was not given, resulting in a final sample of 160 males (mean age = 24.28, *SD* = 3.25 years). All participants were at least 18 years old. In a sensitivity power analysis using G*Power^96^ this sample had sufficient power (> 0.80) to detect an effect size of *r* =  + / − 0.20, assuming one-tailed alpha = 0.05. All procedures were in accordance with relevant guidelines and regulations, and received ethics approval from the local Ethics Committees at the University of Goettingen and the Pennsylvania State University. Informed consent was obtained from all subjects.

### Voice recordings

Standardized video recordings were obtained using a Full-HD camera and Line6 Modell XD-V75 microphones. The participants were instructed to describe what is great about themselves, choosing three domains such as “friendship” or “success in studies/job” from a list of overall eight domains (for details, see^95^). The video clips were cut to a length of 5 s, beginning 5 s after participants had begun to speak, and voice clips were extracted. Five seconds were chosen because vocal parameters usually show strong correlations across different recordings, independent of length and content^88,97^, and both attractiveness and dominance ratings are stable and highly correlated across different recordings^6,97^. Further, the use of relatively brief voice clips allowed us to avoid rater fatigue. The voice clips were analyzed using PRAAT software^98^ (Version 6.0.36). The measures obtained were mean *f*_*o*_, the first four formant frequencies (*F*_1_–*F*_4_), four measures of jitter and five measures of shimmer. Because both jitter (all *r*s > 0.83, *p*s < 0.001) and shimmer measures (all *r*s > 0.56, *p*s < 0.001) were highly intercorrelated, a standardized mean was calculated for each perturbation measure^10^. Additionally, *P*_*f*_ was computed for the first four formants^6^. Formants were measured at each glottal pulse using automated detection in PRAAT. Formant measurement across standardized speech samples produces highly similar results to measurement of individual vowels and averaging across these measurements^6^.

It should be noted that different methods of measuring formant structure are used across studies. Formant dispersion (*D*_*f*_), for example, describes the distance between the highest (e.g., *F*_4_) and lowest formants (e.g., *F*_1_) measured^39^. While *D*_*f*_ is commonly used, it has also been criticized especially for not using information about the middle formants (e.g., *F*_2_ and *F*_3_). Further, although *D*_*f*_ is theoretically dependent on body height, other measures of formant structure have shown stronger relations with body height ^6,33^. One of these measures is formant position (*P*_*f*_) which describes the average standardized formant value for the first *n* formants (e.g., *F*_1_–*F*_4_) and thus utilizes information of all formants measured^6^. Given these advantages of *P*_*f*_ over *D*_*f*_, *P*_*f*_ was chosen as the relevant measure for formant structure in this study. For further discussion, see ^6^.

### Saliva samples

Based on previous studies^99,100^, we controlled for circadian variation in participants’ hormonal reactivity by collecting saliva samples only between 2 and 6 pm. Approximately 12–15 min after each participant arrived at the lab, he rinsed his mouth with water and provided at least 2 ml of saliva via passive drool through a straw, just prior to the video recording. The collected samples were immediately transported to an ultra-low temperature freezer (− 80 °C), where salivary testosterone is expected to be stable for at least 36 months^101^. At the end of the data collection period (see^95^ for details), saliva samples were shipped on dry ice to the Technical University of Dresden and analyzed using chemiluminescence-immuno-assays with high sensitivity (IBL International, Hamburg, Germany). The intra- and inter-assay coefficients (CVs) for cortisol are below 8% and for testosterone below 11%. Basal cortisol and testosterone outliers were identified and winsorized to 3 SDs^102^. To correct for skewness, we log10-transformed both variables.

### Body morphology and strength measurements

As this procedure was also reported in^103^, procedural and methodological descriptions overlap. Participants were scanned three times using a Vitus Smart XXL 3D body scanner, running AnthroScan software (both Human Solutions GmbH, Kaiserslautern, Germany). Participants wore standardized tight underwear and were instructed to stand upright with legs hip-width apart, arms extended and held slightly away from the body, making a fist with thumbs showing forward, the head positioned in accordance with the Frankfort Horizontal, and to breathe normally during the scanning process. Using AnthroScan’s automatic measures (according to ISO 20685), we extracted muscularity-relevant body dimensions from the body scan: body volume, bust-chest girth, buttock girth, chest-to-hip ratio (CHR), forearm girth, lower limb (“leg”) length-to-height ratio (LHR), shoulder-to-hip ratio (SHR), thigh girth, upper arm girth, waist girth, waist-to-chest ratio (WCR), and waist-to-hip ratio (WHR). An aggregate indicator of upper body size was calculated by averaging *z*-standardized shoulder width, bust-chest girth, and upper arm girth^104^. Weight (in kg) was measured as part of the first body scanning process with the integrated SECA 635 scale (SECA, Hamburg, Germany). Body height (in cm) was measured twice using a stadiometer while participants stood barefoot, and the two values were averaged (*ICC* = 0.996). Body-mass index (BMI) was calculated from average weight and height measures (kg/cm^2^). Upper body and handgrip strength were measured using a hand dynamometer (Saehan SH5001). Each measurement was taken three times, starting with handgrip strength, for which participants were asked to use their dominant hand (88.2% used their right). As in^105^, upper body strength was measured by having participants hold the dynamometer in front of their chest with both hands and press both handles toward the middle as strongly as possible. A composite strength measure was formed by averaging the maximum values for each of the three measures of handgrip and upper body strength (*ICC*s: 0.81 and 0.64, respectively).

### Attractiveness and dominance ratings

In exchange for course credit, 120 men (mean age = 19.82, *SD* = 2.71 years) and 120 women (mean age = 19.90, *SD* = 3.80 years) participated in a rating study on short- and long-term attractiveness as well as social and physical dominance at the Pennsylvania State University. All raters were at least 18 years old. Raters were equipped with Sennheiser HD 280 Professional Headphones and seated at private workstations. Raters provided demographic data on age, gender, sexual orientation, and relationship status. To control for the influence of semantic content, we also asked raters to indicate their German language comprehension (“How well do you understand German?”) on a 7-point Likert scale from 0 (“Not at All”) to 6 (“Fluent”). Below, we report results with all participants, but excluding raters score 2 or higher (*n* = 26) does not change results. Raters were then randomly assigned to one of four rating experiments, each asking for perceptions of either short-term attractiveness, long-term attractiveness, social dominance, or physical dominance of 160 randomly assigned voice files (for specific items see Appendix A). The voice file pool contained 320 voice samples that were taken from the 160 former targets before and after the competitive setting^95^. Raters always rated both files of a target, but both recordings of the same individual were separated by at least ten other voice samples. However, only ratings of the recordings before the competition were used in the present study. To ensure that each file was rated 15 times by each sex, a file was removed from the pool of remaining files to be rated once this criterion was met. The only exception was long-term attractiveness, where one male rater dropped out because of technical issues. Because correlations between male and female ratings were high (all *rs* > 0.70, *ps* < 0.001), and intraclass correlations within each rating condition were at least satisfactory (all *ICCs* > 0.76, *ps* < 0.001), mean scores were calculated.

## Results

For tests of directed hypothesis one-tailed tests were used, and for exploratory tests two-tailed tests were used. Analyses were conducted using R^106^.

### Perceptions of Attractiveness and Dominance

**Figure 1:**
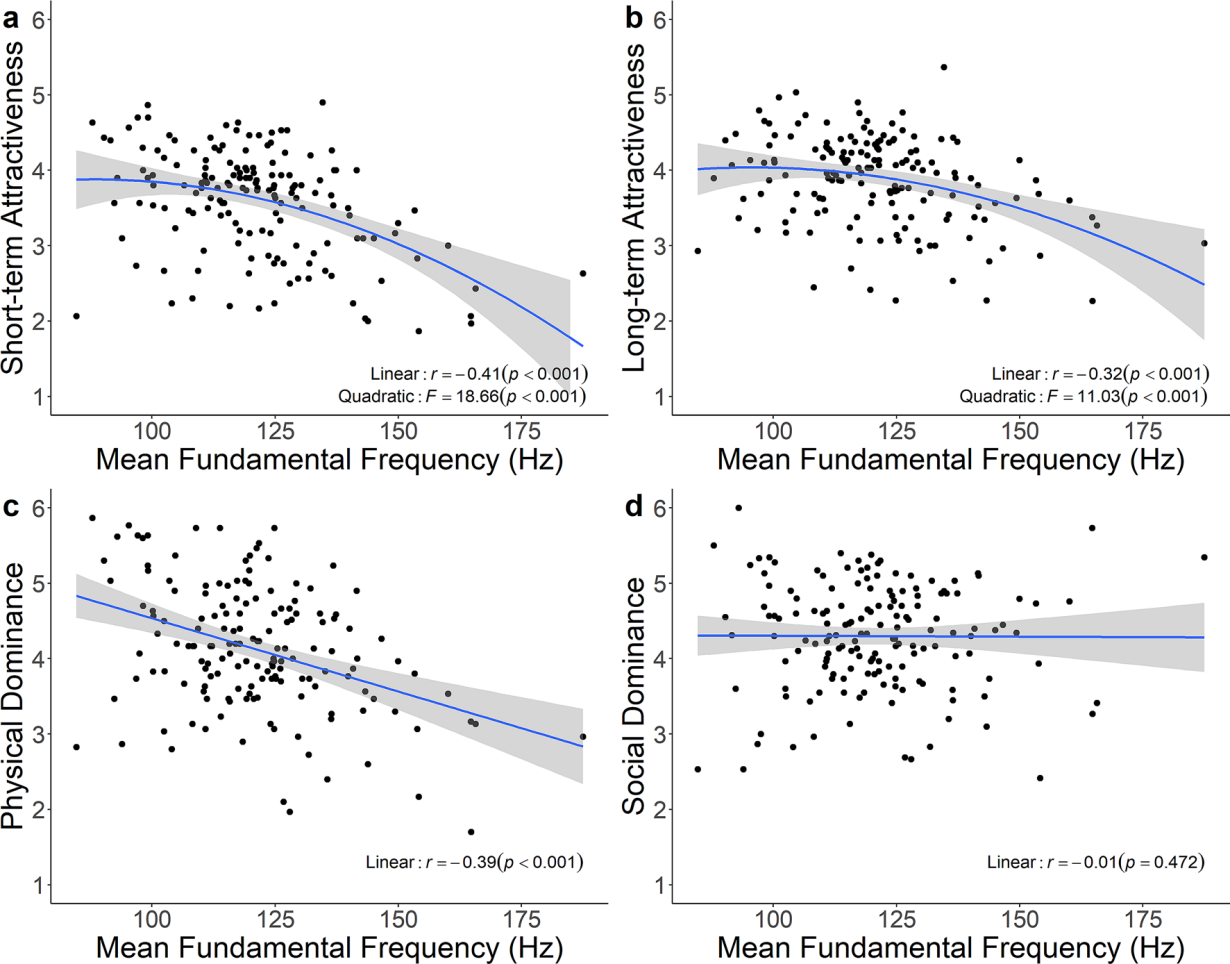
Relationships between male fundamental frequency (*f*_o_) and perceptions. We observed negative curvilinear relationships between *f*_o_ and (**a**) short-term attractiveness and (**b**) long-term attractiveness, (**c**) a negative linear relationship with physical dominance ratings, and (**d**) a non-significant relationship with social dominance ratings. All panels were plotted using the “ggplot2” package^107^.

*Attractiveness* **H1**) Predictions on negative linear and negative quadratic relationships between attractiveness ratings and mean *f*_*o*_ were supported. We found that *f*_*o*_ negatively linearly predicted both short-term and long-term attractiveness. Furthermore, we found significant negatively quadratic (inverted U-shaped) relationships between *f*_*o*_ and both short-term (Fig. 1a) and long-term attractiveness (Fig. 1b). Comparisons of linear and curvilinear models showed that the relationship between *f*_*o*_ and short-term attractiveness was significantly better described by the curvilinear model (*F*_2,157_ = 4.38, *p* = 0.038), while there was no significant difference between models for long-term attractiveness (*F*_2,157_ = 3.76, *p* = 0.054).

**Figure 2:**
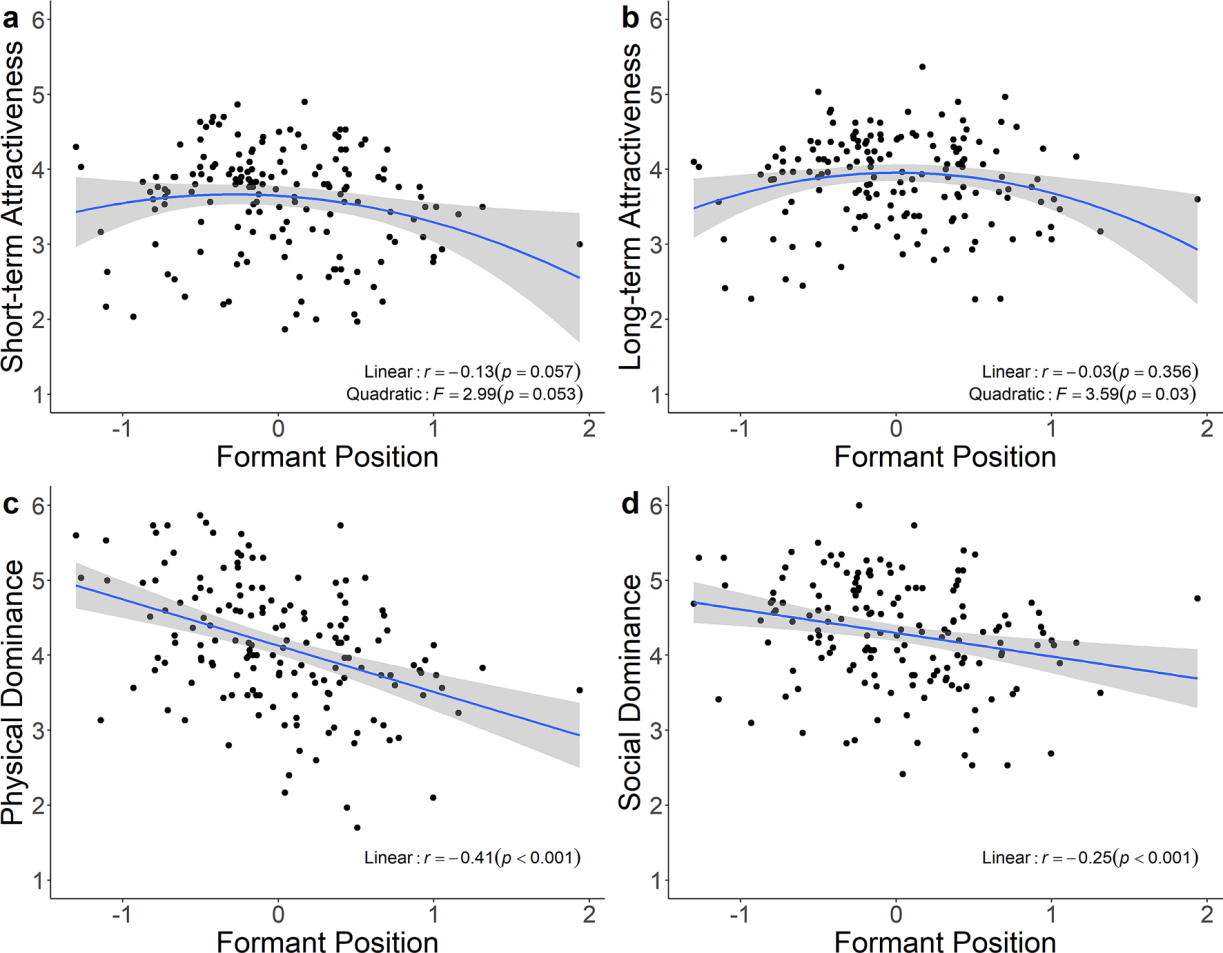
Relationships between male formant position (*P*_*f*_) and perceptions. We observed negative curvilinear relationships between *P*_*f*_ and (**a**) short-term attractiveness and (**b**) long-term attractiveness, (**c**) a negative linear relationship with physical dominance ratings, and (**d**) social dominance ratings. All panels were plotted using the “ggplot2” package^107^.

**H2**) Predictions of negative linear and negative quadratic relationships between attractiveness ratings and *P*_*f*_ were only partially supported. We found no significant linear relationships between *P*_*f*_ and either short-term or long-term attractiveness. While the non-linear relationship of *P*_*f*_ and short-term attractiveness was not significant (Fig. 2a), a significant negative quadratic relationship between *P*_*f*_ and long-term attractiveness emerged (Fig. 2b).

**H3**) The prediction of a stronger relationship between mean *f*_*o*_ and short-term, compared to long-term attractiveness ratings was supported. Although both attractiveness ratings were highly correlated (*r* = 0.82, *p* < 0.001), the relationship between *f*_*o*_ and short-term attractiveness was significantly stronger (*z* = − 2.06, *p* = 0.020) when comparing dependent correlation coefficients^108^.

**H4**) The prediction of a stronger relationship between *P*_*f*_ and short-term, compared to long-term attractiveness ratings was supported; the relationship between *P*_*f*_ and short-term attractiveness was significantly stronger (*z* = − 2.00, *p* = 0.023) when comparing dependent correlation coefficients.

*Dominance* **H5)** The prediction of a negative relationship between dominance ratings and mean *f*_*o*_ was partially supported: *f*_o_ negatively predicted physical dominance (Fig. 1c), but not social dominance ratings (Fig. 1d). **H6**) The prediction of a negative relationship between dominance ratings and *P*_*f*_ was supported. *P*_*f*_ negatively predicted perceptions of both physical (Fig. 2c) and social (Fig. 2d) dominance ratings.

*Independent Predictors* **H7**) The prediction that mean *f*_*o*_ and *P*_*f*_ are independent predictors of attractiveness ratings was partially supported. When *f*_*o*_ and *P*_*f*_ were included in a multiple regression (*F*_2,157_ = 16.78, *p* < 0.001, *R*^2^ = 0.17), *f*_*o*_ negatively predicted short-term attractiveness (*β* = − 0.40, *p* < 0.001), but *P*_*f*_ did not (*β* = − 0.08, *p* = 0.132). Similarly, *f*_*o*_ negatively predicted long-term attractiveness (*β* = − 0.32, *p* < 0.001) in a multiple regression (*F*_2,157_ = 8.94, *p* < 0.001, *R*^2^ = 0.09), but *P*_*f*_ did not (*β* = 0.01, *p* = 0.471). Because the curvilinear relationship between long-term attractiveness and *P*_*f*_ was significant, we investigated whether the linear term of *f*_o_ and the quadratic term of *P*_*f*_ were independent predictors of long-term attractiveness. Indeed, adding the quadratic term of *P*_*f*_ explained significantly more variance in long-term attractiveness ratings (*F*_2,157_ = 3.15, *p* = 0.045), with both predictors remaining significant. **H8**) The prediction that mean *f*_o_ and *P*_*f*_ are independent predictors of dominance ratings was partially supported. Multiple regressions with *f*_o_ and *P*_*f*_ as predictors (*F*_2,157_ = 31.73, *p* < 0.001, *R*^2^ = 0.28) showed that both independently predicted physical dominance (*β* = − 0.35, *p* < 0.001 for *f*_*o*_; *β* = − 0.37, *p* < 0.001 for *P*_*f*_). For social dominance (*F*_2,157_ = 5.12, *p* = 0.007, *R*^2^ = 0.05), *P*_*f*_ was a significant predictor (*β* = − 0.25, *p* < 0.001), but *f*_o_ was not (*β* = 0.02, *p* = 0.391).

### Indirect measures of mate quality and formidability

**Figure 3:**
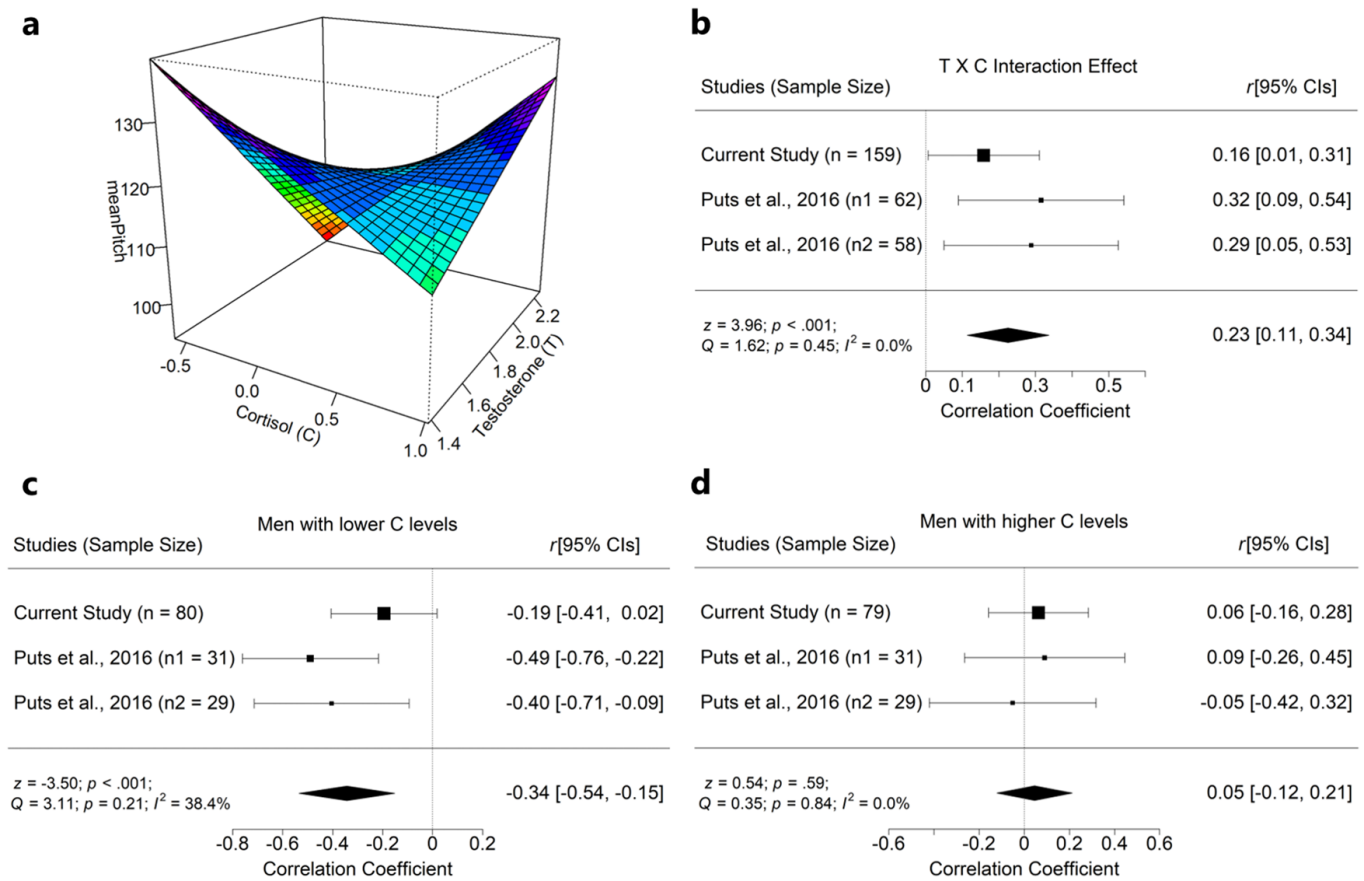
Negative interaction between testosterone and cortisol on male fundamental frequency (*f*_o_). (**a**) A combination of higher testosterone and lower cortisol levels predict lower male *f*_o_ in this study. (**b**) A meta-analysis on the interaction effects across studies, using a random-effects model yielded a significant overall effect. Follow-up meta-analyses on simple slopes of (**c**) lower cortisol levels yielded a significant negative relationship between testosterone and *f*_o_, and (**d**) higher cortisol levels yielded null results. Panel b was plotted via the “rsm” package^109^, and meta-analyses were conducted via the “metaphor” package^110^.

*Testosterone, cortisol and f*_o_ Testosterone levels were not significantly related to *f*_o_ (*r* = − 0.07, *p* = 0.18). However, cortisol and testosterone interacted in predicting *f*_o_ (*β* = 0.16, *p* = 0.024) (Fig. 3a). While these results do not support **H9**) a negative relationship between mean *f*_o_ and testosterone, they supported **H10**) a negative relationship between mean *f*_o_ and testosterone, which is attenuated by high baseline cortisol.

*Body Morphology and P*_*f*_ A significant relationship between *P*_*f*_ and body height was found (*r* = − 0.13, *p* = 0.046), supporting **H11**).

### Exploratory analyses

Strength and *P*_*f*_ Additional exploratory analyses showed significant negative relationships between *P*_*f*_ and strength (*r* = − 0.25, *p* = 0.002). Further, *P*_*f*_ was significantly correlated with multiple body morphology measures related to volume and mass (Table 2).

**Table 2:** Means, standard deviations, and correlations of body morphology measures with P_f_.

Variable	*M*	*SD*	* r*	95% CI
BMI	23.98	3.83	− .23***	[− .37, − .08]
Body volume	79.88	14.03	− .27***	[− .41, − .12]
Bust-chest girth	101.67	8.81	− .29***	[− .43, − .14]
Buttock girth	100.18	7.25	− .26***	[− .40, − .11]
Forearm girth	27.00	1.93	− .28***	[− .42, − .13]
Physical strength	48.40	7.99	− .25**	[− .39, − .09]
Thigh girth	57.58	4.97	− .22**	[− .37, − .07]
Upper body size	56.96	4.13	− .31***	[− .44, − .16]
Upper arm girth	30.20	2.67	− .25**	[− .39, − .09]
Waist girth	84.63	9.86	− .24**	[− .39, − .09]
Weight	78.68	13.96	− .27***	[− .41, − .12]
Chest-to-hip ratio (CHR)	1.02	0.05	− .13	[− .28, .02]
Waist-to-chest ratio (WCR)	1.21	0.07	.03	[− .13, .18]
Waist-to-hip ratio (WHR)	0.84	0.05	− .15	[− .30, .00]
Leg length-to-height ratio (LHR)	0.40	0.01	.12	[− .03, .27]
Shoulder-to-hip ratio (SHR)	0.39	0.02	.08	[− .08, .23]

*M* and *SD* are used to represent mean and standard deviation. Values in square brackets indicate the confidence interval for each correlation. ** indicates *p* < .01; *** indicates *p* < .001.

*Perturbation measures, vocal perception and target parameters* Pearson correlations showed significant negative relationships between shimmer and both social (*r* = − 0.31, *p* < 0.001) and physical dominance (*r* = − 0.31, *p* < 0.001). No significant relationships were found between shimmer and short-term (*r* = − 0.14, *p* = 0.076) or long-term attractiveness (*r* = − 0.12, *p* = 0.122). Jitter showed no significant relationship to any of the four ratings (all *rs* <  + / − 0.11, *ps* > 0.16). Moreover, the only significant relationship between perturbation measures and any of the target parameters was a significant negative correlation between shimmer and baseline cortisol (*r* = − 0.21, *p* = 0.006). Multiple regressions with *f*_o_, *P*_*f*_, jitter and shimmer as predictors and all ratings as outcomes can be found in Tables S1–S4.

*Mediation models *In this analysis (model 7)^111^, cortisol level was recoded into two categories (median split), and their interaction term was computed by multiplying testosterone levels with dichotomized cortisol category. In this model, we found that testosterone levels (*β* =  − 0.09; *p* = 0.321), cortisol category (*β* = 0.07; *p* = 0.367) and their interaction term (*β* = 0.135; *p* = 0.119) did not predict *f*_o_. Adjusting for *P*_*f*_ (*β* =  − 0.39; *p* < 0.001), testosterone (*β* = 0.15; *p* = 0.023) and *f*_o_ (*β* =  − 0.34; *p* < 0.001) significantly predicted physical dominance ratings. The indirect effect of testosterone on dominance ratings via *f*_o_ was not significant (*β* = 0.06; *p* = 0.344), and no significant indirect effect was observed among men with lower cortisol (*β* = 0.04; *p* = 0.227), or men with higher cortisol levels (*β* = 0.02; *p* = 0.832).

**Figure 4:**
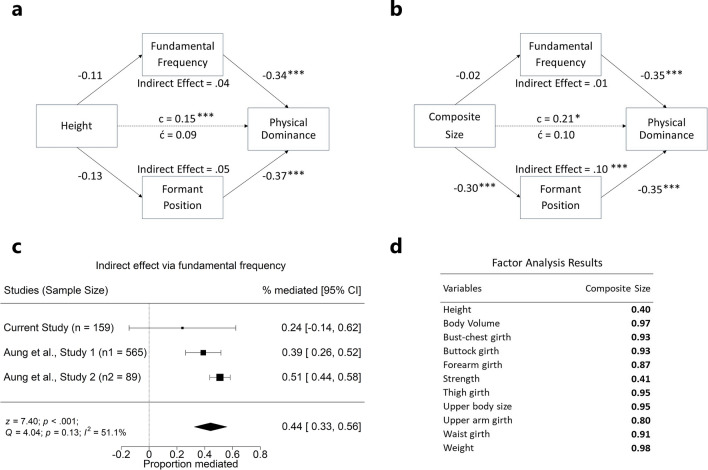
Male fundamental frequency (*f*_o_) and formant position (*P*_*f*_) as mediators of vocalizers’ condition and perceivers’ ratings. (**a**) Although height predicted physical dominance ratings, *f*_o_ and *P*_*f*_ did not mediate this relationship. (**b**) *P*_*f*_, but not *f*_o_, significantly mediated the relationship between composite size and physical dominance ratings. (**c**) Although *f*_o_ was not found to be a significant mediator between height and physical dominance ratings in the present study, a meta-analysis using a random-effects model indicated a significant mediating effect, with *f*_o_ mediating 44% of the relationship between height and physical dominance. Proportion mediated lower than 0 indicates the suppression effect of a mediating variable. In addition, the current study used mean dominance ratings as the primary unit of analyses for calculating proportion mediated, whereas Aung et al., Study 1 (n = 8,103 observations) and Study 2 (n = 6,586 observations) used individual ratings. (d) Using the “nFactors” package^112^ and rotated factors with Varimax method using the “psych” package^113^, we reduced the set of size related measures into one dimensional factor (n = 1), which we labelled “composite size”, via principal axis factoring analysis. ***p < .001.

We ran two additional mediation models: (1) *f*_o_ and *P*_*f*_ were entered as mediators between height and physical dominance ratings, (2) *f*_o_ and *P*_*f*_ were entered as mediators between physical strength and dominance ratings. A composite measure of physical size was extracted from a factor analysis (Fig. 4d) on the following body morphology measures that significantly correlated with *P*_*f*_ (Table 2): height, weight, body volume, bust-chest girth, buttock girth, forearm girth, physical strength, thigh girth, upper body size, upper arm girth, and waist girth. In model 1, *f*_o_ and *P*_*f*_ were entered as mediators between height and physical dominance ratings (Fig. 4a). Neither *f*_o_ nor *P*_*f*_ was a significant mediator. In model 2, we found evidence that *P*_*f*_ mediated the relationship between physical strength condition and physical dominance ratings (Fig. 4b).

*Meta-analyses* We combined results of the present study with prior results^31^ in a meta-analysis to assess the strength of the mediating effect of *f*_o_ on the relationship between height and perceptions of physical dominance. We found a significant overall mediating effect of *f*_o,_ independent of *P*_*f*_ (Fig. 4c); *f*_o_ mediated about 44% the relationship between height and physical dominance ratings.

We also conducted a meta-analysis of the interaction of testosterone and cortisol in predicting *f*_o_. For this analysis, the *t*-value and degrees of freedom (*df*) of the overall interaction effect were transformed into a correlation^114^. The effect of the testosterone and cortisol interaction on male *f*_o_ (*k* = 3, *n* = 279) was significant: *r* = 0.23, *p* = 0.001, 95% CI [0.12, 0.34] (Fig. 3b). In follow-up analyses, the relationship between testosterone and *f*_o_ was significant in men with low cortisol levels (Fig. 3c), but not in those with high cortisol levels (Fig. 3d).

**Figure 5:**
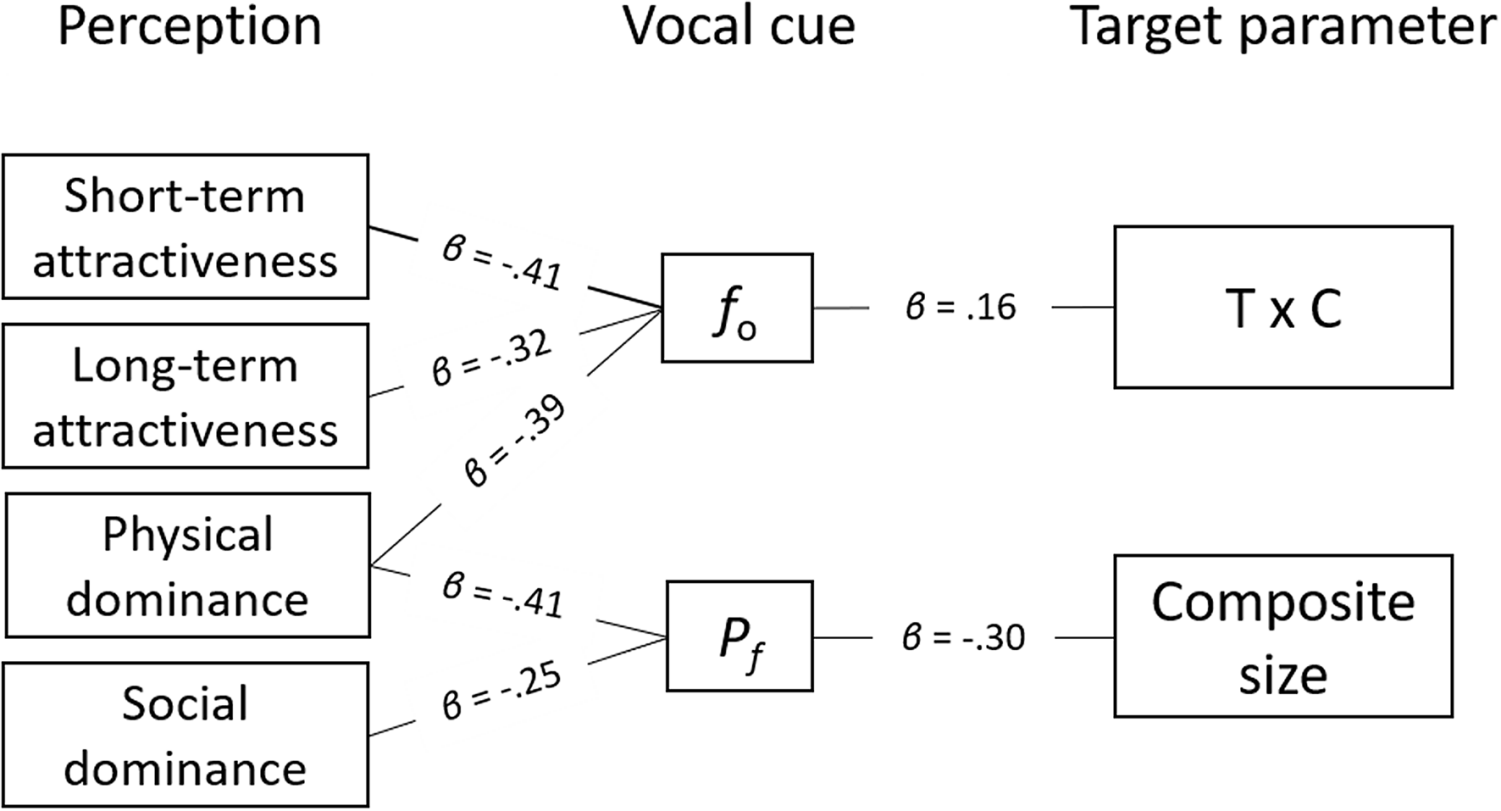
Lens model overview of the study results. Connections indicate significant relations (*p* < .05).

Finally, Fig. 5 provides a lens model^115^ overview of the key relations between perceptions, vocal cues and target parameters found in this study.

## Discussion

We investigated the role of vocal parameters in perceptions of male attractiveness and found that *f*_o_ was the strongest predictor of short- and long-term attractiveness among the vocal parameters measured (*P*_*f*_, shimmer, and jitter). Consistent with previous studies^11,64^, the relationship between *f*_o_ and male vocal attractiveness was both negatively linear and negatively curvilinear, the latter suggesting that women’s voice preferences may reflect a tradeoff between the potential genetic or other benefits versus the potential costs of mating with masculine males^6^. Such costs may include lower investment and perhaps risk of interpersonal violence. Low male *f*_o_ has previously been linked to sexual infidelity^42,88,89,116^, and several lines of evidence suggest that phenotypic masculinity—and vocal masculinity in particular—indicate threat potential not only to same-sex competitors but also to potential mates. For example, images of male-on-female violence disrupted U.S. women’s preferences for both masculine voices and faces^117^, and Colombian women with perceptions of greater local domestic violence preferred less masculine male faces^118^. In another study, Filipino women who were younger and rated themselves as less attractive tended to prefer feminized male *f*_o_, again suggesting that women’s *f*_o_ preferences may in part reflect their own perceived vulnerability^62^. In our data, *f*_o_ was a stronger predictor of short-term than long-term attractiveness, once again supporting the notion of a mate choice trade-off in which putative indicators of genetic fitness are prioritized in short-term contexts, and expected investment and fidelity are prioritized in long-term contexts^90^.

Although *P*_*f*_ predicted strength and body morphology in our study and predicted ratings of attractiveness in some prior studies^13,21^, it did not predict attractiveness in another large sample^5^ and was unrelated to short-term attractiveness and only weakly negatively curvilinearly linked to long-term attractiveness in the present study. These lines of evidence suggest that the information provided by formant frequencies may be less relevant to mate quality than that provided by *f*_o_. By contrast, shimmer negatively predicted both short- and long-term attractiveness ratings. Shimmer is utilized to assess vocal quality in clinical contexts, such that pathological voices show higher shimmer levels than those of healthy individuals^119–121^; however, a composite of shimmer and harmonics-to-noise ratio (which were highly correlated) showed no relationship to dominance or attractiveness perception in a recent study^5^. These divergent findings may be explained by the fact that the latter study used voice samples in which male individuals read a standardized voice passage, while our study used more natural but less standardized stimuli that might have been influenced more strongly by the speaker’s affective state.

Importantly, a Fisherian mate choice model via runaway sexual selection has also been suggested as a possible driver favoring low male *f*_o_^14,122^. A Fisherian model would suggest that female choice primarily drives and exaggerates the evolution of male traits; hence, the model predicts that females prefer males with the lowest *f*_o_. However, evidence from the current study and previous studies^15,62,65^ (suggests a general preference for lower *f*_o_ by women, but also a relatively stronger negative linear relationship between *f*_o_ and dominance perceptions by men across studies^29^.

While *f*_o_ predicted both short- and long-term attractiveness, it predicted physical dominance but not social dominance, in line with previous studies^13,123^. *P*_*f*_ and shimmer were linked to both social and physical dominance ratings. A possible explanation for this pattern of results is that social dominance is influenced less by threat potential and more by other qualities, such as competence, communication and cooperation skills, or leadership qualities. These attributes might be more strongly associated with *P*_*f*_ and shimmer than with *f*_o_.

The other aim of this study was to explore whether attention to vocal cues is adaptive by investigating the information content of acoustic parameters. We replicated a negative relationship between *P*_*f*_ and height^33^ and found that *P*_*f*_ negatively predicted strength and several body morphology measures. Men with lower *P*_*f*_ were taller, stronger, and had larger bodies in general. Further, our mediation analysis indicated that *P*_*f*_, independently of *f*_o_, mediated the relationship between a composite measure of body size and physical dominance ratings.

Importantly, baseline cortisol and testosterone levels interacted in predicting *f*_o_, such that testosterone levels more strongly negatively predicted *f*_o_ as cortisol levels decreased across participants. When we entered the interaction term between testosterone and median-split cortisol levels into our exploratory moderated mediation analyses, the interaction effect became non-significant, likely due to reduced statistical power^124^ from dichotomizing a continuous variable (cortisol). Nevertheless, the overall interaction between testosterone and cortisol in predicting male *f*_o_ was confirmed in a meta-analysis (Fig. 3b). Male *f*_o_ was negatively correlated with testosterone when cortisol was low, whereas no significant relationship was observed between male *f*_o_ and testosterone when cortisol was high (Fig. 3c). These patterns of relationships may help clarify why dose-dependent effects of androgen levels on the intensity of elaborate male traits are sometimes undetected^125^, and why *f*_o_ is only weakly correlated with testosterone when cortisol is not considered. Across a variety of species, testosterone and cortisol are linked to measures of physical condition, including disease, stress, and diet^126^. The interaction between testosterone and cortisol, in particular, has been tied to immune function in birds^127^, but the functional and behavioral correlates of this hormonal interaction in humans are not yet clear^36,128^, and most studies are arguably underpowered. Further, a recent meta-analysis found only modest support for an interactive relationship between testosterone and cortisol in predicting status-relevant behavior (e.g., dominance & risk taking) and suggested that this association could be driven by publication bias and flexibility in data analysis^129,130^. Although only one paper^5^ besides the current one has reported the specific interaction effect of testosterone and cortisol on male *f*_o_, the meta-analysis reported here suggests that the interaction is robust.

There is widespread agreement^5,11,27,40,46^ that low male *f*_o_ evolved to exaggerate apparent size by leveraging a predisposition to perceive low frequencies as emanating from large sound sources. Phylogenetic reconstruction suggests that relatively male *f*_o_ evolved in the common ancestor of the catarrhine primates after their divergence from platyrrhines approximately 43.5mya^5^. Given the weak correspondence between *f*_o_ and body size, some have argued that *f*_o_ is purely deceptive and is not an honest indicator of physical dominance^27,28,131^. Others have suggested that *f*_o_ may reliably correlate with other salient speaker characteristics such as status, threat, and dominance, and that these dimensions may overlap with, and hence intrude onto impressions of, size^46^. Our results better comport with the latter possibility. Indeed, relatively low male *f*_o_ tends to be lost in primate species in which male-male mating competition is reduced, suggesting that there are costs associated with low *f*_o_ that cause this trait to be selected against when compensatory benefits are absent.

Deference to males with low *f*_o_ is demonstrably costly in humans in terms of social status, mates, and reproduction, and thus attention to *f*_o_ would seemingly be selected against if *f*_o_ did not provide valid information about male condition^30^. However, this does not mean that *f*_o_ is cheat-proof, or that the assessment of condition or formidability from *f*_o_ is largely accurate. Honest signals are often corrupted into conventional signals where cheating is common because the assessment of the signal itself is costly to the receiver^132^. Although we did not find support for the cortisol-moderated mediation role of *f*_o_ between testosterone levels and physical dominance ratings in the present sample, this may be explained by reduced statistical power due to dichotomized cortisol levels and reduced sample sizes for testing two separate indirect effects. Indeed, we found a strong meta-analytic support for an overall interaction between testosterone and cortisol in predicting male *f*_o_, suggesting that *f*_o_ conveys underlying endocrine state, if imprecisely, and lower male *f*_o_ has consistently been shown to predict perceptions of physical dominance across multiple studies. Likewise, a recent study^31^ reported that *f*_o_ mediated the relationship between developmental condition (measured via height) and physical dominance ratings in two separate samples with different types of vocal stimuli. Although we did not find that *f*_o_ significantly mediated the relationship between height and physical dominance ratings in our data, our meta-analysis suggests that *f*_o_ mediates about 44% of the relationship between height and physical dominance ratings. Collectively, our findings support the hypothesis that, while the correlation between *f*_o_ and underlying quality is imperfect, *f*_o_ might be utilized as one of many cues for assessing competitors and potential mates^29^ because it communicates the quality of the signaler significantly better than chance^132,133^.

Shimmer also negatively predicted social and physical dominance ratings, as well as lower cortisol levels. The latter finding is consistent with prior evidence that shimmer is reduced when stress is induced experimentally or when the speaker is under high tension^134^. However, the other perturbation measure, jitter, showed no such associations. Future research should continue to explore the relevance of jitter and shimmer to human sexual selection (see also), as they have been shown to be associated with pathological voice quality^120^ and body shape in men^41^ and might therefore be relevant in contexts of sexual selection.

One limitation with our study is that we tested only hypotheses associated with receiver-independent costs and did not consider receiver-dependent costs associated with attention to male *f*_o_. Some^135,136^ have suggested that additional mechanisms that incorporate receiver-dependent costs are required to ensure signal honesty. For example, under a mating-motive priming condition, male voices with low *f*_o_ enhanced recognition for men with high threat potential^135^ and elicited aggressive cognitions and intent in men who perceived themselves to be more dominant and stronger^136^. Future studies should investigate the extent to which receiver-dependent and independent costs are needed in ensuring the signal honesty of low *f*_o_ in cross-cultural contexts.

Following suggestions by Lakens^137^, we used one-sided significance tests for preregistered directional hypotheses. The only result influenced by this decision is the relation between *P*_*f*_ and height, which would be non-significant using a two-sided test. However, we note that meta-analytic findings^33^ suggest a robust link between *P*_*f*_ and height, and the lack of a significant relation in this particular study is likely due to a lack of statistical power. Thus, also our conclusions remain highly similar when two-sided tests are used.

## Conclusion

Vocal parameters were linked to hormone levels, as well as body morphology and physical strength, and appear to be used for judgements relevant to intrasexual competition and intersexual mate choice. The present study thus provides evidence that natural interindividual variation in men’s vocal parameters influences judgements of attractiveness and dominance because these parameters provide valid information about speakers’ underlying condition.

### Supplementary information

Supplementary information.
