# Author Correction: Linking human male vocal parameters to perceptions, body morphology, strength and hormonal profiles in contexts of sexual selection

**DOI:** 10.1038/s41598-021-89157-9

**Published:** 2021-05-03

**Authors:** Christoph Schild, Toe Aung, Tobias L. Kordsmeyer, Rodrigo A. Cardenas, David A. Puts, Lars Penke

**Affiliations:** 1 Department of Psychology, University of Copenhagen, Øster Farimagsgade 2A, 1353 Copenhagen, Denmark; 2 Department of Anthropology and Center for Brain, Behavior and Cognition, Pennsylvania State University, University Park, PA 16802 USA; 3 Department of Psychology and Leibniz ScienceCampus Primate Cognition, University of Goettingen, Gosslerstrasse 14, 37073 Göttingen, Germany; 4 Department of Psychology, Pennsylvania State University, University Park, PA 16802 USA

Correction to: *Scientific Reports* https://doi.org/10.1038/s41598-020-77940-z, published online 04 December 2020

The original version of this Article contained an error in Figure 3, where the *p* value for the *z*-test was incorrect in panel (d). The original Figure 3 and accompanying legend appears below.

**Figure 3:**
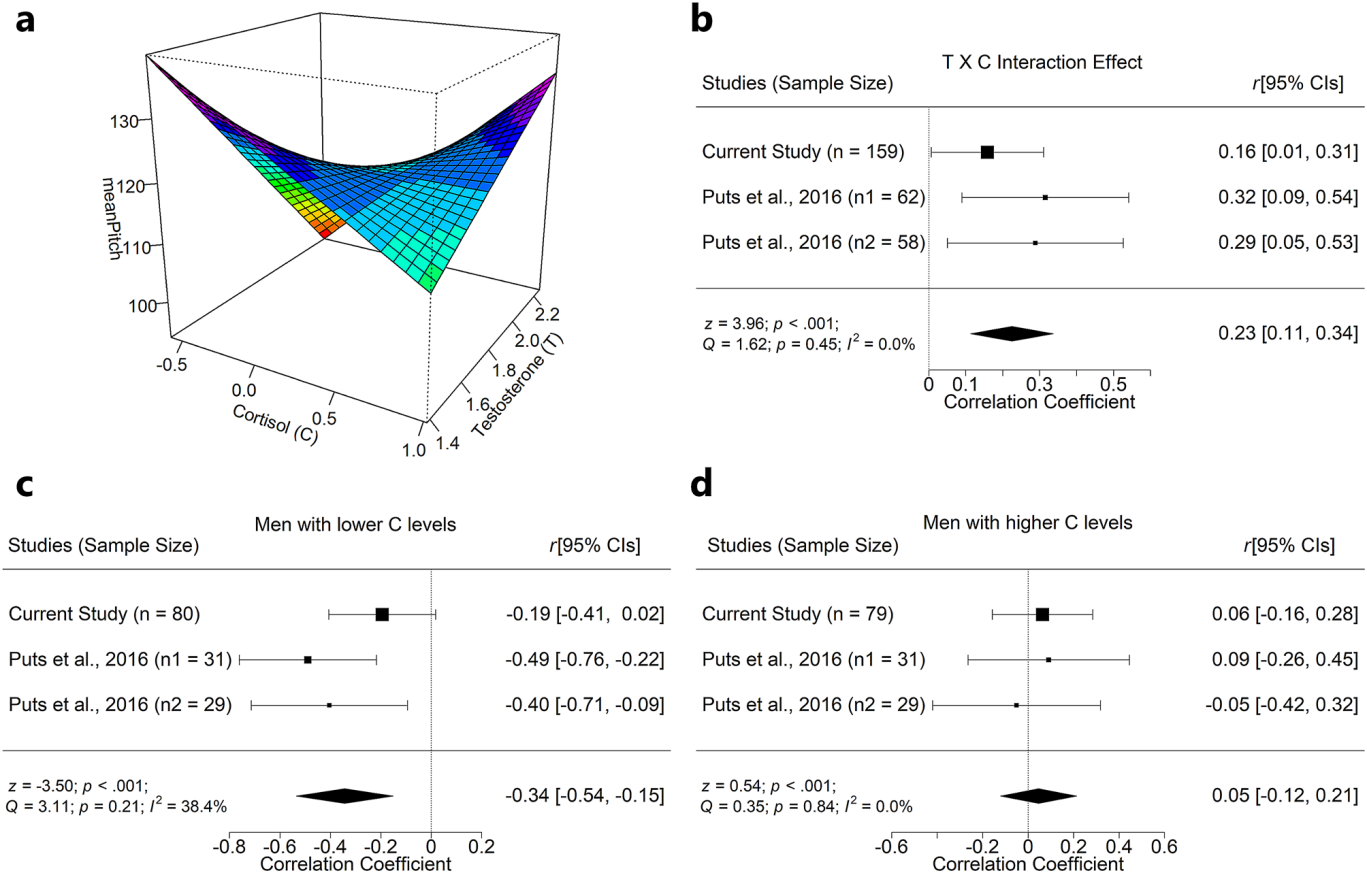
Negative interaction between testosterone and cortisol on male fundamental frequency (*f*_o_). (**a**) A combination of higher testosterone and lower cortisol levels predict lower male *f*_o_ in this study. (**b**) A meta-analysis on the interaction effects across studies, using a random-effects model yielded a significant overall effect. Follow-up meta-analyses on simple slopes of (**c**) lower cortisol levels yielded a significant negative relationship between testosterone and *f*_o_, and (**d**) higher cortisol levels yielded null results. Panel b was plotted via the “rsm” package^109^, and meta-analyses were conducted via the “metaphor” package^110^.

The original Article has been corrected.

