# Supplementary material for: Linking human male vocal parameters to perceptions, body morphology, strength and hormonal profiles in contexts of sexual selection
Source: Sci Rep. 2020 Dec 4;10:21296. doi: 10.1038/s41598-020-77940-z (PMC7719159; doi:10.1038/s41598-020-77940-z)
Supplement: Supplementary file 1 — Supplementary information. [file 41598_2020_77940_MOESM1_ESM.docx]

**Linking human male vocal parameters to perceptions, body morphology, strength and hormonal profiles in contexts of sexual selection**

Christoph Schild^1^, Toe Aung^2^, Tobias L. Kordsmeyer^3^, David A. Puts^2^, Rodrigo A. Cardenas^4^, & Lars Penke^3^

^1^ Department of Psychology

University of Copenhagen

Øster Farimagsgade 2A, 1353 Copenhagen, Denmark

^2^ Department of Anthropology & Center for Brain, Behavior and Cognition

Pennsylvania State University

University Park, PA 16802, USA

^3^ Department of Psychology & Leibniz ScienceCampus Primate Cognition

University of Goettingen

Gosslerstrasse 14, 37073 Goettingen, Germany

^4^ Department of Psychology

Pennsylvania State University

University Park, PA 16802, USA

Corresponding author: Christoph Schild (cs@psy.ku.dk)

Table S1

*Multiple Regression of Short-term Attractiveness on Vocal Parameters*

| Predictor | *b* | *b*  95% CI  [LL, UL] | *beta* | *beta*  95% CI  [LL, UL] | *sr^2^* | *sr^2^*  95% CI  [LL, UL] | *r* | Fit |
| --- | --- | --- | --- | --- | --- | --- | --- | --- |
| (Intercept) | 5.55** | [4.81, 6.29] |  |  |  |  |  |  |
| F0 | -0.02** | [-0.02, -0.01] | -0.39 | [-0.53, -0.25] | .14 | [.05, .24] | -.41** |  |
| Pf | -0.06 | [-0.24, 0.12] | -0.05 | [-0.20, 0.10] | .00 | [-.01, .02] | -.13 |  |
| Jitter | 0.10 | [-0.02, 0.22] | 0.14 | [-0.03, 0.30] | .01 | [-.02, .05] | .10 |  |
| Shimmer | -0.16* | [-0.28, -0.03] | -0.21 | [-0.37, -0.04] | .03 | [-.02, .08] | -.14 |  |
|  |  |  |  |  |  |  |  | *R^2^*  = .209** |
|  |  |  |  |  |  |  |  | 95% CI[.09,.30] |
|  |  |  |  |  |  |  |  |  |

*Note.* A significant *b*-weight indicates the beta-weight and semi-partial correlation are also significant. *b* represents unstandardized regression weights. *beta* indicates the standardized regression weights. *sr^2^* represents the semi-partial correlation squared. *r* represents the zero-order correlation. *LL* and *UL* indicate the lower and upper limits of a confidence interval, respectively.
*: *p* < .05, **: *p* < .01.

Table S2

*Multiple Regression of Long-term Attractiveness on Vocal Parameters*

| Predictor | *b* | *b*  95% CI  [LL, UL] | *beta* | *beta*  95% CI  [LL, UL] | *sr^2^* | *sr^2^*  95% CI  [LL, UL] | *r* | Fit |
| --- | --- | --- | --- | --- | --- | --- | --- | --- |
| (Intercept) | 5.18** | [4.52, 5.83] |  |  |  |  |  |  |
| F0 | -0.01** | [-0.02, -0.01] | -0.30 | [-0.45, -0.15] | .09 | [.01, .17] | -.32** |  |
| Pf | 0.04 | [-0.12, 0.20] | 0.04 | [-0.11, 0.19] | .00 | [-.01, .01] | -.03 |  |
| Jitter | 0.10 | [-0.01, 0.20] | 0.16 | [-0.01, 0.33] | .02 | [-.02, .06] | .11 |  |
| Shimmer | -0.14* | [-0.25, -0.03] | -0.22 | [-0.39, -0.04] | .03 | [-.02, .09] | -.12 |  |
|  |  |  |  |  |  |  |  | *R^2^*  = .140** |
|  |  |  |  |  |  |  |  | 95% CI[.04,.22] |
|  |  |  |  |  |  |  |  |  |

*Note.* A significant *b*-weight indicates the beta-weight and semi-partial correlation are also significant. *b* represents unstandardized regression weights. *beta* indicates the standardized regression weights. *sr^2^* represents the semi-partial correlation squared. *r* represents the zero-order correlation. *LL* and *UL* indicate the lower and upper limits of a confidence interval, respectively.
* indicates *p* < .05. ** indicates *p* < .01.

Table S3

*Multiple Regression of Physical Dominance on Vocal Parameters*

| Predictor | *b* | *b*  95% CI  [LL, UL] | *beta* | *beta*  95% CI  [LL, UL] | *sr^2^* | *sr^2^*  95% CI  [LL, UL] | *r* | Fit |
| --- | --- | --- | --- | --- | --- | --- | --- | --- |
| (Intercept) | 6.21** | [5.41, 7.00] |  |  |  |  |  |  |
| F0 | -0.02** | [-0.02, -0.01] | -0.34 | [-0.47, -0.21] | .11 | [.03, .19] | -.39** |  |
| Pf | -0.47** | [-0.67, -0.28] | -0.32 | [-0.45, -0.18] | .09 | [.02, .17] | -.41** |  |
| Jitter | 0.10 | [-0.02, 0.23] | 0.12 | [-0.03, 0.27] | .01 | [-.01, .04] | -.00 |  |
| Shimmer | -0.28** | [-0.42, -0.15] | -0.31 | [-0.46, -0.16] | .07 | [.01, .14] | -.31** |  |
|  |  |  |  |  |  |  |  | *R^2^*  = .360** |
|  |  |  |  |  |  |  |  | 95% CI[.23,.45] |
|  |  |  |  |  |  |  |  |  |

*Note.* A significant *b*-weight indicates the beta-weight and semi-partial correlation are also significant. *b* represents unstandardized regression weights. *beta* indicates the standardized regression weights. *sr^2^* represents the semi-partial correlation squared. *r* represents the zero-order correlation. *LL* and *UL* indicate the lower and upper limits of a confidence interval, respectively.
* indicates *p* < .05. ** indicates *p* < .01.

Table S4

*Multiple Regression of Social Dominance on Vocal Parameters*

| Predictor | *b* | *b*  95% CI  [LL, UL] | *beta* | *beta*  95% CI  [LL, UL] | *sr^2^* | *sr^2^*  95% CI  [LL, UL] | *r* | Fit |
| --- | --- | --- | --- | --- | --- | --- | --- | --- |
| (Intercept) | 4.14** | [3.36, 4.91] |  |  |  |  |  |  |
| F0 | 0.00 | [-0.01, 0.01] | 0.03 | [-0.12, 0.18] | .00 | [-.01, .01] | -.01 |  |
| Pf | -0.24* | [-0.43, -0.05] | -0.19 | [-0.34, -0.04] | .03 | [-.02, .08] | -.25** |  |
| Jitter | 0.12 | [-0.01, 0.24] | 0.16 | [-0.01, 0.33] | .02 | [-.02, .06] | -.03 |  |
| Shimmer | -0.26** | [-0.39, -0.13] | -0.34 | [-0.51, -0.17] | .09 | [.01, .17] | -.31** |  |
|  |  |  |  |  |  |  |  | *R^2^*  = .149** |
|  |  |  |  |  |  |  |  | 95% CI[.05,.23] |
|  |  |  |  |  |  |  |  |  |

*Note.* A significant *b*-weight indicates the beta-weight and semi-partial correlation are also significant. *b* represents unstandardized regression weights. *beta* indicates the standardized regression weights. *sr^2^* represents the semi-partial correlation squared. *r* represents the zero-order correlation. *LL* and *UL* indicate the lower and upper limits of a confidence interval, respectively.
* indicates *p* < .05. ** indicates *p* < .01
